# Neoadjuvant chemoradiotherapy plus tislelizumab followed by surgery for esophageal carcinoma (CRISEC study): the protocol of a prospective, single-arm, phase II trial

**DOI:** 10.1186/s12885-023-10687-8

**Published:** 2023-03-15

**Authors:** Jinsong Yang, Ai Huang, Kunyu Yang, Ke Jiang

**Affiliations:** 1 Cancer Center, Union Hospital, Tongji Medical College, Huazhong University of Science and Technology, Wuhan, 430023 China; 2 Department of Thoracic Surgery, Union Hospital, Tongji Medical College, Huazhong University of Science and Technology, Wuhan, 430022 China

**Keywords:** Esophageal carcinoma, Neoadjuvant, Chemoradiotherapy, Immunotherapy, Tislelizumab, Surgery, Pathologic complete response, Phase II trial

## Abstract

**Background:**

The failure rate after neoadjuvant chemoradiotherapy followed by surgery is approximately 34.6%–48% for resectable esophageal carcinoma. Pathologic complete response after neoadjuvant chemoradiotherapy is an important factor in predicting lower recurrence and better survival. Whether the sequential addition of immunotherapy to neoadjuvant chemoradiotherapy will be beneficial to improving the pathologic complete response rate is unknown.

**Methods:**

Patients with pathologically confirmed thoracic esophageal squamous cell carcinoma and at clinical T1-2N1-3M0 or T3-4aN0-3M0 (stage II–IVA) according to the eighth edition of American Joint Committee on Cancer staging will be allocated to receive neoadjuvant radiotherapy (41.4 Gy with 23 fractions to planning target volume) with concurrent chemotherapy (albumin-bound paclitaxel, 100 mg/m^2^, once weekly for five weeks; carboplatin, area under the curve of 2 mg/mL/min, once weekly for five weeks) plus tislelizumab monotherapy sequentially (200 mg every three weeks for three cycles, beginning from the first to the 14th day after the end of radiotherapy). Then, subtotal esophagectomy with two-field lymphadenectomy, including the whole mediastinum and abdomen, will be performed. The primary endpoint for this study is the pathologic complete response rate after neoadjuvant chemoradiotherapy plus tislelizumab.

**Discussion:**

The optimal timing of the combination of immunotherapy and neoadjuvant chemoradiotherapy in esophageal carcinoma is not determined. The results of this phase II trial will be helpful to clarify the safety and efficacy of the sequential addition of tislelizumab after neoadjuvant chemoradiotherapy for locally advanced resectable esophageal carcinoma.

**Trial registration:**

This study was approved on January 26, 2021 and retrospectively registered with ClinicalTrials.gov (NCT04776590) on March 1, 2021.

## Background

Esophageal cancer is the seventh most common malignant tumor and the sixth leading cause of cancer death in the world [1]. Surgical resection is the mainstay for locally advanced esophageal squamous cell carcinoma (ESCC), but the rate of radical resection (R0 resection) is only approximately 69%–93%, and the 5-year overall survival rate after surgery is only 34%–42% [2–4]. The overall recurrence rate after radical surgery is as high as 48%–57.1% [5–8], most of which are locoregional recurrences (LRR) accounting for 83.6% and distant recurrence (DR), accounting for 23.7% [7].

Neoadjuvant therapy aims to decrease preoperative staging and increase R0 resection rate to reduce tumor relapse thereby improving survival. In terms of improving overall survival (OS) and decreasing recurrence with good safety for locally advanced resectable thoracic ESCC in the CROSS [4] and NEOCRTEC5010 studies [9], nCRT followed by surgery was recommended by both the NCCN [10] and ASCO [11] esophageal cancer guidelines as a preferred treatment option. However, the strategy of nCRT plus surgery does not resolve all the problems. The 5–10-year overall failure rate after nCRT followed by surgery was as high as 34.6%–48.0% [12, 13]. It is known that pathologic complete response (pCR) after nCRT plus surgery predict a significantly lower recurrence rate and higher OS rate compared with non-pCR [14–18]. Thus, efforts to increase pCR rate should be made before surgery to improve postoperative clinical outcome for ESCC patients.

Programmed cell death protein-1 (PD-1), as an immune checkpoint, is a negative costimulatory receptor, which is inducibly expressed primarily on the surface of activated T cells. The interaction between PD-1 and its ligands, PD-L1 and PD-L2, inhibits T cell response to antigen stimulating, which results in the exhaustion of T cells and attenuates the antitumor effect. Thus, blocking the binding of PD-1 and its ligands by immune checkpoint inhibitors assists in the recovery of T cells activity and the promotion of immune clearance against cancer cells. In recent years, anti-PD-1(L1) immunotherapy has revolutionized the landscape of treatment in many cancers, especially in advanced or metastatic esophageal cancer [19–23]. However, for locally advanced resectable ESCC, the role of immunotherapy and the optimal combination of immunotherapy with nCRT in the setting of neoadjuvant therapy have not been clarified. Considering the success of durvalumab in the PACIFIC study [24] and the failure of avelumab in the JAVELIN Head and Neck 100 study [25], sequential administration of PD-1(L1) inhibitors after chemoradiotherapy seems to be a rational option. An exploratory analysis (NCT02125461, unpublished) revealed that patients with non-small cell lung cancer receiving durvalumab within 14 days from the last radiotherapy had a better objective response rate (ORR), progression-free survival (PFS), and OS than patients receiving durvalumab after 14 days from the last radiotherapy, which implied that the timing of PD-1(L1) inhibitors joining in chemoradiotherapy is important. We assumed that early sequential administration of tislelizumab after nCRT would mostly benefit ESCC patients. Thus, we launched this prospective, single-arm, phase II study aiming to assess the safety and efficacy of nCRT plus early sequential administration of tislelizumab followed by surgery for resectable thoracic ESCC.

## Methods/design

### Study design

**Fig. 1:**
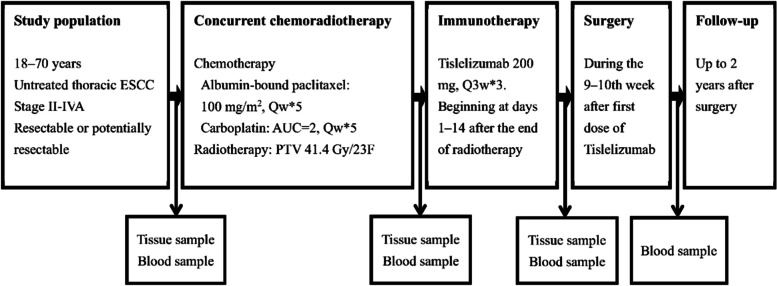
Study design

This single-arm, phase II study aims to evaluate the safety and efficacy of sequential tislelizumab after nCRT followed by surgery in resectable locally advanced ESCC. The primary endpoint of this study is the pCR rate. The secondary endpoints include safety, major pathological response (MPR) rate, R0 resection rate, quality of life, failure rate, disease-free survival (DFS), and OS. Collection of tumor tissues and peripheral blood samples are planned for exploratory biomarker analysis at several timepoints (before chemoradiotherapy, after chemoradiotherapy and before tislelizumab, before surgery, and after surgery) (Fig. 1). The study started on January 26, 2021 and the planned closing date is December 31, 2024.

### Study procedures

All subjects who signed informed consent forms will be allocated to nCRT plus sequential tislelizumab followed by surgery.

First, the subjects will receive computed tomography (CT) simulation scanning with contrast and a thickness of 5 mm in the supine position. Gross tumor volume (GTV) is defined as primary esophageal cancer and locoregional metastatic lymph nodes shown in physical examination, CT with contrast, esophageal radiography, electronic gastroscopy, and positron emission tomography-CT. Both the elective nodal irradiation (ENI) and the involved-field irradiation (IFI) methods will be allowed for the delineation of the clinical target volume (CTV). The ENI method is as follows: CTV encompasses a proximal and distal margin of 3 cm and a 0.5–1.0 cm radial margin around the GTV. For upper and middle thoracic esophageal cancer, CTV further includes the supraclavicular region and superior mediastinum. For lower thoracic esophageal cancer, CTV further includes the inferior mediastinum and celiac trunk region. The IFI method is as follows: CTV encompasses a proximal and distal margin of 1–2 cm and a 0.5–1.0 cm radial margin around the GTV. The planning target volume (PTV) is defined as a three-dimensional 5-mm outer margin of the CTV. External beam radiation with the intensity-modulated radiotherapy or volumetric modulated arc therapy technique will be adopted for physical planning. The dose for organs at risk is as low as possible. A total radiation dose of 41.4 Gy will be prescribed for PTV and delivered in 23 fractions of 1.8 Gy per fraction, five fractions per week, using a linear accelerator with 6–8 MV X-rays. Concurrent chemotherapy will be administered with intravenous infusion of albumin-bound paclitaxel (100 mg/m^2^, weekly) and carboplatin (area under curve = 2, weekly) five times, beginning on the first or the second day of radiotherapy.

Then, tislelizumab will be intravenously administered from the first to the 14th day after the end of radiotherapy with a dose of 200 mg every three weeks for three cycles.

In the end, the subjects will receive subtotal esophagectomy with two-field lymphadenectomy including the whole mediastinum and abdomen during the ninth to 10th week after the beginning of tislelizumab. Both McKeown esophagectomy and Ivor Lewis esophagectomy will be allowed. Whatever the location of the primary tumor, video-assisted thoracoscopic resection with a transthoracic approach will be preferred.

### Eligible participants

#### Inclusion criteria

1. Aged 18–70 years old and of any gender.
2. Untreated thoracic esophageal carcinoma.
3. Pathologically confirmed ESCC.
4. Clinical T1-2N1-3M0 or T3-4aN0-3M0 (II-IVA) according to the eighth edition of American Joint Committee on Cancer (AJCC) staging.
5. Resectable or potentially resectable according to clinical evaluation.
6. Karnofsky’s performance status score of ≥ 70.
7. Sufficient organ function is expected to be tolerable to surgery and chemoradiotherapy. Permitted blood routine test (no blood transfusion within 14 days) results: a. hemoglobin ≥ 100 g/L. b. neutrophil count ≥ 1.5 × 10^9^/L. c. platelet count ≥ 100 × 10^9^/L. Blood biochemistry examinations show normal bilirubin, alanine aminotransferase, aspartate aminotransferase, creatinine, and urea nitrogen.
8. Pregnancy test (women of childbearing age) was negative at the time of screening.
9. Fertile men and women of childbearing age must agree to use contraception throughout the study period.
10. Able to obey study protocol and sign informed consent.

#### Exclusion criteria

1. Current or former patients with other cancers in the latest 5 years, not including cured carcinoma in situ of the cervix, nonmelanoma skin cancer, and superficial bladder tumor.
2. Definite bleeding tendency or significant bleeding symptoms, including but not limited to gastrointestinal bleeding and nasal bleeding within 28 days before enrollment, or persistent hemorrhagic diseases or coagulation disorders.
3. Suffering from autoimmune diseases.
4. Unstable angina pectoris and/or congestive heart failure or vascular disease requiring hospitalization within 12 months before enrollment (e.g., aortic aneurysm or peripheral venous thrombosis requiring surgical repair), or other cardiac damage that may affect the safety evaluation of the study drug judged by investigators (e.g., poorly controlled arrhythmia, myocardial infarction, or ischemia).
5. Subject has a history of abdominal fistula, digestive tract perforation, abdominal abscess, or acute gastrointestinal hemorrhage within 6 months before enrollment.
6. The presence of aggravated chronic obstructive pulmonary disease or other respiratory system diseases requiring hospitalization within 28 days before enrollment.
7. Active pulmonary infection and/or acute bacterial, viral, or fungal infection requiring intravenous antibiotic treatment within 28 days prior to enrollment.
8. Pregnant or nursing mothers.
9. HBsAg positive and peripheral blood hepatitis B virus DNA level of more than 1 × 10^3^ copies/L.
10. Positive anti-hepatitis C virus antibody.
11. Positive anti-human immunodeficiency virus antibody.
12. Received systemic immunomodulator treatment (including but not limited to interferon and interleukin-2) within 28 days before enrollment.
13. Participated in clinical trials of other antitumor therapy within 28 days before enrollment.
14. Previously received antitumor therapy for esophageal cancer.
15. Previous surgical history leading to the inability to perform a gastrointestinal reconstruction.
16. X-ray imaging showed signs of perforation tendency, such as deep and large niche, sharp thorn, angulation, and torsion of the esophagus, within 28 days before enrollment.
17. Cancer progression was found before administration of tislelizumab.
18. The presence of interstitial pneumonia of grade II or above at the end of chemoradiotherapy; liver and kidney function injury of grade III or above, or decreased hemoglobin, neutrophils, or platelets count of grade IV or above failing to recover to grade 1 or below within 2 weeks after radiotherapy.
19. Subjects known to be allergic to tislelizumab.

### Pathologic examination

The surgery specimens will be macroscopically and microscopically reviewed and recorded by a team of experienced pathologists using the standard protocol [26], including primary tumors, lymph nodes, margins, and treatment effect. The tumor site will be thoroughly sampled with submission of the entire tumor bed for specimens without grossly obvious residual tumor. The response of the primary tumor will be evaluated according to a modified Ryan scheme for tumor regression score [26]: 0 (*complete response*): no viable cancer cells; 1 (*near-complete response*): single cells or rare small groups of cancer cells; 2 (*partial response*): residual cancer with evident tumor regression, but more than single cells or rare small groups of cancer cells; and 3 (*poor or no response*): extensive residual cancer with no evident tumor regression. The number of lymph nodes detected should be 15 or more. pCR is defined as the absence of residual cancer cells within the primary esophageal tumor and dissected lymph nodes (ypT0N0M0). MPR is defined as < 10% residual viable tumor remaining in posttherapy specimen [27]. Pathologic stage will be classified according to the eighth edition of AJCC staging.

### Outcome measures

Clinical tumor response evaluation will be according to Response Evaluation Criteria in Solid Tumor (RESIST) version 1.1. All patients will receive a thorough workup before enrollment (Table 1). Esophageal radiography, cervical, chest, and upper abdomen CT with contrast will be performed for clinical tumor response evaluation after the end of radiotherapy and before the beginning of tislelizumab, before surgery. After surgery, esophageal radiography, cervical, chest, and upper abdomen CT with contrast will be repeated every 3–6 months for treatment failure evaluation until 2 years after enrollment.

**Table 1:** Workup before treatment

Workup before treatment
•Karnofsky’s performance status score
•EORTC cancer quality of life scale QLQ-C30 (V3.0) and QLQ-OES18
•Height, body weight, and vital signs
•Blood, urine, and stool routine test. Serum biochemistry test, including bilirubin, alanine aminotransferase, aspartate aminotransferase, creatinine, urea nitrogen, electrolyte, Lactate dehydrogenase, troponin I, erythrocyte sedimentation rate, C-reactive protein, lipase, and amylase
•Blood cytokines and lymphocyte subsets
•Electrocardiogram and echocardiography
•Electronic gastroscopy, esophageal tumor tissue biopsy, esophageal endoscopic ultrasonography (EUS), esophageal radiography, cervical, and chest and upper abdomen CT with contrast; nuclear bone imaging
•Whole body positron emission tomography-CT and brain nuclear magnetic resonance imaging with contrast are optional

Adverse reactions will be evaluated and recorded weekly according to the Common Terminology Criteria for Adverse Events Version 5.0 from the beginning of radiotherapy until one month after surgery. Quality of life will be assessed weekly using the EORTC-QLQ C30 (V3.0) and EORTC-OES-18 scales after enrollment and every 6–12 weeks after surgery.

Treatment failure includes locoregional recurrence and distant metastasis. DFS is defined as the interval from R0 resection to treatment failure or death or last follow-up. OS is defined as the interval from the beginning of radiotherapy to death of any cause or last follow-up.

### Statistics

We calculated the sample size according to the design of a single-stage phase II clinical trial. The pCR rate after nCRT was set as 27.7% for locally advanced ESCC which was reported in a multicenter, phase III trial in 2021 in China [28]. We hypothesized that the expected pCR rate would be 52.7% after nCRT plus sequential tislelizumab, which meant that an improvement of 25% or more of the pCR rate would be achieved. Then, 24 subjects are needed at an α-level of 0.05 (one-sided) and power of 80%. Considering dropouts at a rate of 20%, 30 subjects in total should be enrolled.

The descriptive statistical method will be applied to the presentation of baseline characteristics, toxicities, measures of effectiveness, and quality of life. The Kaplan–Meier method will be used to calculate survival rates. The chi-squared test and Mann–Whitney *U* test will be used for the comparison of potential subgroups.

## Discussion

The optimal neoadjuvant therapy for locally advanced resectable ESCC has been studied and debated for more than half a century. Both neoadjuvant radiotherapy and chemotherapy have been studied for decades in locally advanced thoracic ESCC. Despite the increase in R0 resection rate and survival benefit shown in some studies, no widely accepted conclusion has been established that either neoadjuvant radiotherapy [29] or neoadjuvant chemotherapy (nCT) [30] alone could improve overall survival. However, this situation changed after concurrent chemoradiotherapy was successfully applied to the neoadjuvant therapy for locally advanced thoracic ESCC in the CROSS [4] and NEOCRTEC5010 studies [9].

The CROSS study [4] randomized resectable esophageal or esophagogastric-junction cancer patients (clinical stage T1N1M0 or T2-3N0-1M0 according to the sixth edition of the Union for International Cancer Control [UICC] classification) to the surgery alone group or the nCRT followed by surgery group. The R0 resection rate was significantly higher in the nCRT group than in the surgery alone group (92% vs. 69%, *P* < 0.001). In addition, the pCR rate was 49% in the nCRT group. Postoperative complications were comparable in the two treatment groups. OS was significantly better in the nCRT group than that in the surgery alone group (5-year OS rate, 47% vs. 34%, *P* = 0.003). The 10-year outcome of the trial [13] demonstrated that the effect of nCRT on OS was not time-dependent and the absolute 10-year OS benefit was 13%. A significantly less LRR with (13% vs. 22%) or without (8% vs. 18%) synchronous DR developed in the nCRT group than in the surgery alone group. Another phase III trial, the NEOCRTEC5010 study [9], enrolled 451 patients with potentially resectable thoracic ESCC (clinically T1-4N1M0/T4N0M0 according to the sixth edition of the UICC staging) who were randomly assigned to nCRT followed by the surgery and surgery alone groups. The pCR rate was 43.2% in the nCRT group. Compared with the surgery alone group, the nCRT group had a higher R0 resection rate (98.4% vs. 91.2%, *P* = 0.002), a better OS (median, 100.1 months vs. 66.5 months, *P* = 0.025), and a prolonged DFS (median, 100.1 months vs. 41.7 months, *P* = 0.001). The most common grade 3 or 4 adverse events during nCRT were leukopenia (48.9%) and neutropenia (45.7%). The nCRT group had a significantly decreased 5-year cumulative LRR (15.3% vs. 27.9%), DR (24.3% vs. 40.1%), and overall recurrence (32.2% vs. 50.9%) rates compared with the surgery alone group.

For nCT, which was mainly recommended in Japan based on the JCOG9907 study [31], the conclusion was controversial and the strength of evidence was not enough [32]. A meta-analysis [30] revealed that ESCC did not benefit from nCT in terms of all-cause mortality. Recently, the JCOG1109 NExT study [33] reported that nCT with three cycles of docetaxel/cisplatin/fluorouracil (DCF) had a significantly higher 3-year OS rate (72.1% vs. 62.6%, *P* = 0.006) than nCT with two cycles of cisplatin/fluorouracil (CF) for locally advanced ESCC, while there was no significant difference in the 3-year OS between the nCRT and nCT groups with two cycles of CF. However, febrile neutropenia in the DCF group was higher than that in the CF group (16.3% vs. 1.0%) during neoadjuvant therapy. Unfortunately, there was no direct comparison of survival between the neoadjuvant DCF and nCRT groups in the study design [34]. A phase III study [28] in China reported that nCRT followed by surgery had similar safety and better pCR rate (27.7% vs. 2.9%; *P* < 0.001) than nCT followed by surgery for the treatment of locally advanced ESCC, while the survival result as the primary endpoint was immature. A population-based study [35] comprising 2,367 patients with ESCC demonstrated that the nCRT group had significantly higher pCR rate (50.9% vs. 30.4%; *P* < 0.001), R0 resection rate (92.8% vs. 82.4%; *P* < 0.001), and 5-year OS rate (45.0% vs. 38.0%, *P* = 0.026) than the nCT group. Thus, nCRT should be a more effective option for neoadjuvant therapy in locally advanced ESCC.

Tislelizumab is a human IgG4 monoclonal antibody that binds to and blocks the PD-1 receptor expressed on activated immune cells, including T lymphocytes [36]. Its efficacy and safety have been tested in several clinical trials. The global phase III study, RATIONALE-302 [37], demonstrated that tislelizumab monotherapy as a second-line treatment achieved a significant improvement in OS compared with chemotherapy (median OS, 8.6 months vs. 6.3 months, *P* = 0.0001) for pretreated advanced or metastatic ESCC. Tislelizumab resulted in a higher ORR (20.3% vs. 9.8%) and longer median duration of response (DoR) (7.1 months vs. 4.0 months) versus chemotherapy. Patients treated with tislelizumab had fewer treatment-related adverse events (TRAEs) (73.3% vs. 93.8%) and ≥ grade 3 TRAEs (18.8% vs. 55.8%) versus chemotherapy. Tislelizumab plus chemotherapy as first-line therapy for advanced or metastatic ESCC is being evaluated in the global double-blind, phase 3, RATIONALE-306 study. The interim analysis results (NCT03783442, unpublished) show a significant improvement with tislelizumab plus chemotherapy in OS (Median OS, 17.2 months vs. 10.6 months, *P* < 0.0001) and PFS (Median PFS, 7.3 months vs. 5.6 months, *P* < 0.0001) compared with placebo plus chemotherapy. OS benefit with tislelizumab plus chemotherapy was observed regardless of baseline PD-L1 expression level and consistently across prespecified subgroups. Tumor response was greater (ORR, 63.5% vs. 42.4%) and more durable (DoR, 7.1 months vs. 5.7 months) with tislelizumab plus chemotherapy. Incidences of the most common adverse events were similar between the treatment groups. Based on the results of the RATIONALE-302 and RATIONALE-306 studies, the antitumor activity of tislelizumab for advanced or metastatic ESCC was confirmed in second-line treatment and promising in first-line treatment.

Similarly, other PD-1 antibodies, such as nivolumab, pembrolizumab, camrelizumab, and sintilimab, demonstrated consistently that they as the second-line monotherapy antibodies significantly improved OS compared with the investigator-chosen chemotherapy in the overall group or subgroup population of pretreated advanced or metastatic ESCC [38–41]. The combination of each of these antibodies with platinum-containing chemotherapy significantly improved OS and PFS compared with chemotherapy alone in the overall group as the first-line therapy for advanced or metastatic ESCC [19–21, 23].

In the setting of locally advanced resectable thoracic ESCC, the combination of PD-1(L1) antibodies with chemotherapy or chemoradiotherapy as neoadjuvant therapy was under investigation in some phase II studies, most of which adopted chemotherapy plus immunotherapy as neoadjuvant therapy. The reported overall pCR rate after nCT combined with immunotherapy ranged from 17%–50% [42–45], while the overall pCR rate after nCRT and immunotherapy achieved up to 55.6% [46]. The results of some of these studies are close to the pCR level of 43%–49% in the CROSS [3] and NEOCRTEC5010 [9] studies. However, a real-world multicenter retrospective study [47] in China showed that the total pCR (ypT0N0) rate was 25.5% for nCT plus immunotherapy and 42.3% for nCRT plus immunotherapy, and the overall R0 resection rate was 97.7% with the addition of immunotherapy to neoadjuvant therapy. TRAEs were observed in 53.8% patients with low severity (grade 1–2, 39.2%; grade 3–4, 13.2%; and grade 5, 1.4%). These results suggest that anti-PD-1 immunotherapy may be safe in combination with nCT and nCRT for ESCC, while the most effective combination is to be further studied.

Radiotherapy is widely used to control tumor by breaking the DNA double-strand and subsequently causing cancer cell death. In addition to the local therapeutic effect in the irradiated tumor, radiotherapy has a systemic influence on the host, which has been traditionally considered to be immunosuppressive since the high radiosensitivity of lymphocytes and rapid decrease in peripheral blood lymphocytes count after conventional radiotherapy. However, accumulating preclinical and clinical data [48–51] suggest that radiotherapy could produce immunostimulating effects by promoting in situ vaccination with the release of tumor-associated antigens, altering the cancer cell immunophenotype, recruiting and activating dendritic cells, and promoting T-cell infiltration into the tumor microenvironment. Ultimately, these effects of radiotherapy may induce immunogenic cell death of cancer cells and sensitize refractory tumors toward immunotherapy [52]. In contrast, some clinical studies [53–57] have revealed that the expression level of PD-L1 was elevated after radiotherapy and high expression of PD-L1 was associated with poor prognosis in ESCC. Therefore, the increased expression of PD-L1 on tumor cells or infiltrating immune cells after radiotherapy has to be counteracted by blocking the PD-1 pathway, which objectively creates an opportunity for the administration of immunotherapy. As a successful precedent, the survival benefit from such a combination of durvalumab following chemoradiotherapy has been demonstrated in the PACIFIC study [24] for locally advanced non-small cell lung cancer. In contrast, another PD-L1 inhibitor avelumab, which was administered before, during, and after the chemoradiotherapy phase in the JAVELIN Head and Neck 100 study [25], did not improve either PFS or OS compared with the placebo for locally advanced squamous cell carcinoma of the head and neck. One possible reason inferred to was the depletion of T cells after definite chemoradiotherapy. Therefore, sequential administration of immunotherapy after radiotherapy seems to be a rational choice. Furthermore, an exploratory analysis (NCT02125461, unpublished) in the PACIFIC study revealed that patients receiving durvalumab within 14 days from the last radiotherapy achieved a better ORR, PFS, and OS than patients receiving durvalumab after 14 days from the last radiotherapy, which implied that the optimal timepoint of PD-1(L1) inhibitors combined with chemoradiotherapy seems to be early rather than late after the end of radiotherapy.

In summary, given the extensive application and significant progress of PD-1 inhibitors in recent years, the incorporation of immunotherapy into nCRT of ESCC seems to be an inevitable trend. However, the optimal timing of the combination of immunotherapy with nCRT in ESCC is not determined. The results of the CRISEC study will provide novel evidence for neoadjuvant immunotherapy in locally advanced resectable thoracic ESCC.

## Data Availability

The datasets used and/or analyzed during the current study are available from the corresponding author upon reasonable request.

## Abbreviations

AJCC: American Joint Committee on Cancer
CF: Cisplatin/fluorouracil
CRISEC: Chemoradiotherapy plus tislelizumab followed by surgery for esophageal carcinoma
CT: Computed tomography
CTV: Clinical target volume
DCF: Docetaxel/cisplatin/fluorouracil
DFS: Disease-free survival
DR: Distant recurrence
ENI: Elective nodal irradiation
ESCC: Esophageal squamous cell carcinoma
EUS: Endoscopic ultrasonography
GTV: Gross tumor volume
IFI: Involved-field irradiation
LRR: Locoregional recurrences
MPR: Major pathological response
nCT: Neoadjuvant chemotherapy
ORR: Objective response rate
OS: Overall survival
pCR: Pathologic complete response
PD-1: Programmed cell death protein-1
PFS: Progression-free survival
PTV: Planning target volume
RESIST: Response Evaluation Criteria in Solid Tumor
TRAE: Treatment-related adverse event
UICC: Union for International Cancer Control
